# Inter-Individual Differences in the Initial 80 Minutes of Motor Learning of Handrim Wheelchair Propulsion

**DOI:** 10.1371/journal.pone.0089729

**Published:** 2014-02-21

**Authors:** Riemer J. K. Vegter, Claudine J. Lamoth, Sonja de Groot, Dirkjan H. E. J. Veeger, Lucas H. V. van der Woude

**Affiliations:** 1 University of Groningen, University Medical Center Groningen, Center for Human Movement Sciences, Groningen, The Netherlands; 2 Amsterdam Rehabilitation Research Center | Reade, Amsterdam, The Netherlands; 3 Faculty of Human Movement Sciences, Research Institute MOVE, Vrije Universiteit, Amsterdam, The Netherlands; 4 Faculty of Mechanical, Maritime and Materials Engineering**,** section Biomechatronics & Biorobotics, Delft University of Technology, Delft, The Netherlands; 5 University of Groningen, University Medical Center Groningen, Center for Rehabilitation, Groningen, The Netherlands; University of Sao Paulo, Brazil

## Abstract

Handrim wheelchair propulsion is a cyclic skill that needs to be learned during rehabilitation. Yet it is unclear how inter-individual differences in motor learning impact wheelchair propulsion practice. Therefore we studied how early-identified motor learning styles in novice able-bodied participants impact the outcome of a low-intensity wheelchair-practice intervention. Over a 12-minute pre-test, 39 participants were split in two groups based on a relative 10% increase in mechanical efficiency. Following the pretest the participants continued one of four different low-intensity wheelchair practice interventions, yet all performed in the same trial-setup with a total 80-minute dose at 1.11 m/s at 0.20 W/kg. Instead of focusing on the effect of the different interventions, we focused on differences in motor learning between participants over the intervention. Twenty-six participants started the pretest with a lower mechanical efficiency and a less optimal propulsion technique, but showed a fast improvement during the first 12 minutes and this effect continued over the 80 minutes of practice. Eventually these initially fast improvers benefitted more from the given practice indicated by a better propulsion technique (like reduced frequency and increased stroke angle) and a higher mechanical efficiency. The initially fast improvers also had a higher intra-individual variability in the pre and posttest, which possibly relates to the increased motor learning of the initially fast improvers. Further exploration of the common characteristics of different types of learners will help to better tailor rehabilitation to the needs of wheelchair-dependent persons and improve our understanding of cyclic motor learning processes.

## Introduction

Handrim wheelchair propulsion is a cyclic bimanual form of ambulation that needs to be learned during early rehabilitation by people with a lower-limb disability. Compared to other forms of ambulation the gross mechanical efficiency of handrim propulsion, i.e. the ratio of external power output over internal power production is low, while at the same time overuse problems are common [1]–[5]. Yet, different intervention studies have shown that, through low-intensity practice both mechanical efficiency and propulsion technique of handrim wheelchair propulsion can improve, possibly reducing the load on the wheelchair user [6]–[13]. However, it is unknown how inter-individual differences in motor learning impact the outcomes of wheelchair propulsion practice in such an early stage.

Within the rehabilitation environment, using the International Classification of Functioning, Disability & Health (ICF) framework, there is appreciation for inter-individual differences in outcomes of health and disability [14]. An important domain in this framework is ‘personal factors’ such as age, gender, physical ability, self-efficacy and motivational level [15]. Other important personal factors related to motor learning are trainability and talent, i.e. the individual response to exercise [16], [17] and the ability to adopt and optimize motor skills [18], [19]. For instance, inter-individual differences were found in the effect of regular physical activity on maximal oxygen consumption, submaximal heart rate response, cholesterol and systolic blood pressure [20]. Correlations of these variables with age, gender or ethnic background were low. In contrast, baseline values of heart rate and blood pressure strongly correlated with the effect of the intervention. Individuals with higher baseline values and thus a worse physical condition showed larger reductions in heart rate and blood pressure due to training [20].

Analogous to exercise programs that focus on improving physical capacity, low-intensity practice sessions aim to improve the motor skill of individuals. On a group level it has been shown that inexperienced individuals improve their wheelchair propulsion skills through practice [6]–[13]. Yet, this improvement over the group may not fully apply to each member of that group. Although there is increasing evidence of inter-individual differences in learning a new motor task, this notion is still rarely assessed [18], [21]–[24].

Not only between, but also within individuals, human movement is intrinsically variable [25], [26]. This intra-individual movement variability can for instance be found between limbs performing the same action (i.e. interlimb variability), or in one limb repeating a cyclic movement over time (i.e. intralimb variability). Such variability is assumed to not only be the reflection of noise and/or error, but also to be functional and to contain features that may provide insight in motor learning [27]–[29] and pathological processes [30]–[34]. From this perspective, intra-individual variability is seen as a mechanism allowing individuals to adapt their movements as a function of organismic, environmental and task constraints [35], [36]. Variability allows the performer to explore different motor solutions, facilitating the discovery and adoption of individualized optimal patterns of coordination, possibly reducing the energetic cost [37]. In the current study, changes in the intra-individual variation in learning wheelchair propulsion are studied based on the coefficient of variation (CV) defined as the percentage standard deviation of the mean of a given technique parameter.

Because of several unique features, the study of handrim wheelchair propulsion is suitable to gain insight into inter- and intra-individual differences in early motor learning processes of cyclic motor tasks in novice able-bodied individuals. Firstly, wheelchair propulsion is cyclic, which makes it possible to evaluate steady-state submaximal performance using energy consumption and thus mechanical efficiency as a generic outcome measure of motor learning [38]. Secondly, the movement is sufficiently unconstrained to allow for performance of the task in different ways, allowing propulsion technique to change between the left and right wheel and over time within one side [39], [40]. Finally, for most people, wheelchair propulsion is a new task. Therefore, in the study of motor learning, learning wheelchair propulsion is highly suitable as a model to study the initial acquisition of a cyclic skill. Wheelchair skill acquisition in early rehabilitation can well be studied with able-bodied participants, thus reducing heterogeneity within the participant group, which might be expected in for instance a group of participants with a spinal cord injury due to the level and completeness of the lesion, health history or upper-body asymmetries beyond age, gender and training status [41]. On another note, researchers do not have to burden patients early on as they are learning to cope with the far-reaching effects of a new SCI.

In our previous work, early inter-individual motor learning differences were found in 70 novice able-bodied wheelchair users [42]. Two different groups were formed based on a relative 10% increase in mechanical efficiency between the 4^th^ and 12^th^ minute of practice. The Initially Slow Improvers (ISI) already demonstrated a significantly higher mechanical efficiency and more skilled propulsion technique at the first steady-state measurement (the 4^th^ minute) compared to the Initially Fast Improvers (IFI). However, the ISI did not further increase in proficiency in the next 8 minutes, whereas the IFI, despite starting at a lower level of mechanical efficiency, were able to improve in mechanical efficiency each next trial. After 12 min of practice the groups showed a similar absolute level of mechanical efficiency [42].

For rehabilitation it is important to know how these short-term inter-individual differences in motor learning impact the outcome of an intervention over a longer timescale. From the 70 participants in the previously discussed twelve-minute study, 39 continued in four different low-intensity interventions. Instead of focusing on the effect of the different intervention types, the main aim of the current study is to follow the two designated motor learning groups (ISI/IFI) over time, to find out whether their initially different motor learning styles still differed after 80 min practice.

The research question of the current study is therefore: how do early-identified motor learning styles among two different groups of able-bodied novice participants impact the outcome of an 80 min low-intensity wheelchair-practice intervention? The early motor learning differences will again be assessed during the 12-minute pretest based on a relative increase of either less or equal to 10% or higher than 10% in gross mechanical efficiency [42]. The two identified groups will then be analyzed over the pre -and post-test to see how the early differences between the groups impact the eventual intervention outcome.

It is hypothesized from earlier work [42] that the same types of early differences in motor learning between individuals will be present over the follow-up period. Also, it is hypothesized that the mean outcomes of both groups shall differ in the coefficient of variation, showing differences in the variability of task execution between the groups. These initial motor learning differences are expected to impact the final outcome of the intervention, where those participants that learn more in the pretest are expected to be the ones who benefit most from the given practice [20].

## Methods

### Participants

After written informed consent was provided, 39 able-bodied men spread over four experimental groups fulfilled our criteria for participation in this study (table 1). To compare our results with previous research the criteria for inclusion were male, between 18–65 years, no prior experience in wheelchair propulsion, and absence of any medical contra-indications [6], [8], [9], [43], [44]. The study was approved by the Local Ethics Committee, of the Center for Human Movement Sciences, University Medical Center Groningen, University of Groningen, the Netherlands.

**Table 1:** Personal characteristics of the four practice groups: One–day monotonous (ODM), three-week monotonous (TWM), one-day seat-height (ODS) and one-day feedback (ODF).

Study	Duration	Dose:	Test-nature	Age(yrs)	Height(m)	Weight(kg)	n-total	n-IFI	n-ISI
ODM	1-day	80 min	Monotonous	22.0 (2.0)	1.89 (0.11)	81.1 (19.4)	10	8	2
TWM	3-week	80 min	Monotonous	22.8 (3.9)	1.89 (0.07)	83.8 (11.6)	13	8	5
ODS	1-day	80 min	Variable(seat height)	23.3 (2.6)	1.86 (0.07)	80.4 (14.0)	10	6	4
ODF	1-day	80 min	Variable(feedback)	23.3 (4.1)	1.84 (0.04)	74.8 (6.1)	6	4	2
**total**				22.9 (3.2)	1.87 (0.07)	80.0 (12.8)	39	26	13

ISI are the ISI, i.e. the participants that increased less or equal to 10% in mechanical efficiency.

IFI are the IFI, i.e. the participants that increased more then 10% in mechanical efficiency.

### Protocol

**Figure 1:**
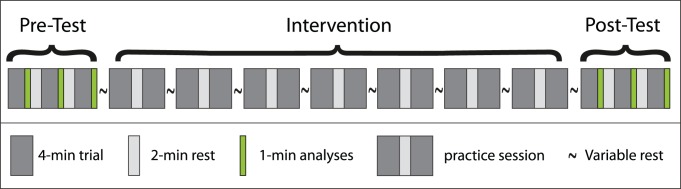
Setup of practice. A practice session consists of two 4-min trials separated by 2-min rest at 0.20 W/kg. The pre and post-test had one trial extra and the last minute of each trial was used for analyses. The time between practice sessions (variable rest) was either 30 min or 48 hr.

Each of the 39 participants were involved in one of four intervention formats. The four wheelchair interventions were different in nature (table 1), but were performed in the same experimental and trial-setup (figure 1) and had the same dose of 80 min propulsion at a relative power output of 0.20 W/kg. Although the four intervention studies had a common design, allowing the combination of the data at a more global level (see Statistics), each intervention had their own question beyond the main aim of the present study (manuscripts under preparation). The low intensity was chosen to minimize fatigue or training effects and focus primarily on motor learning. The first key difference between the interventions was the time-scale over which the 80 min practice was performed; the participants either participated in a single-day or a three-week experiment. During the single-day experiment the intervention shown in figure 1 was completed in one continuous experiment with 30 min rest between each 8-min practice session, whereas during the three-week experiment each 8-min practice session was separated by 48 hours. The second key difference was practice variation. One single-day group (ODM) and one three-week group (TWM) trained monotonously during the intervention. Two other single-day studies trained with variations. Participants in the first study practiced with four different absolute seat-heights as provided by the experimental wheelchair. The seat-height counterbalanced over the 7 blocks of the intervention (ODS). The participants of the second single-day study received real-time feedback (ODF) on seven propulsion technique variables, also in a counterbalanced order, earlier described by Richter et al. [45]. These seven propulsion technique characteristics were individually presented as a bar graph on a monitor in each of the seven practice blocks. Participants were free to use this feedback, but never got any specific instruction on how to manipulate any of these parameters. Thus for all groups technique improvements over time are assumed to have occurred as a function of practice.

Eventually, n = 26 participants were identified as IFI and n = 13 were ISI.

### Experimental Setup

All trials were performed on a level treadmill of 2.4 m length by 1.2 m width (Forcelink b.v, Culemborg The Netherlands) in the same experimental wheelchair (Double Performance BV, Gouda, The Netherlands) with 24-inch measurement wheels. Each participant practiced according to the schedule presented in figure 1. The first 40 seconds of a trial were used to get the treadmill up to a speed of 1.11 m/s (4 km/h). The required power output to get to 0.20 W/kg was imposed by adding mass to a pulley system. For each participant a drag test was performed prior to the start of the experiment. Based on the calculated drag force of the wheelchair-user combination at the required constant speed of the treadmill (1.11 m/s) and the participants’ body mass, the added mass to the pulley was calculated [4], [46]. For data analysis the last minute of each trial in the pre-test and post-test were used (i.e. trial 1, 2, 3 and 18, 19, 20).

### Measurement Wheels

One standardized experimental wheelchair was used and no individual adjustments were made for individual participants. The regular rear wheels of the standardized wheelchair were replaced with two instrumented wheels; on the left the Optipush (MAX Mobility, LLC, Antioch, TN, USA) and on the right the Smartwheel (Three Rivers Holdings, Mesa, AZ, USA). Both wheels measure 3-dimensional forces and torques applied to the handrim, combined with the angle under which the wheel is rotated. Data were wirelessly transferred to a laptop at 200 Hz (Optipush) and 240 Hz (Smartwheel). Both wheels were synchronized by an electronic pulse at the start of each measurement [40]. Data from the Optipush were primarily used in the analyses, only when the Optipush data were lacking they were replaced with Smartwheel data after mirroring those data. Time averaged data of both wheels attached to the left and right side of the same wheelchair placed on a treadmill showed good comparability, with an intra-class correlation (ICC) of 0.89 for mean power output and ICC’s higher than 0.90 for propulsion technique characteristics [40]. Therefore, the time averaged outcomes of the left and right wheel in this experiment were assumed to be comparable.

### Energy Expenditure

Oxygen consumption (VO2) was continuously measured during each practice session using breath-by-breath open circuit spirometry (Oxycon Pro-Delta, Jaeger, Hoechberg, Germany). The gas analyzer was calibrated using a Jaeger 5 l syringe, room air and a calibration gas mixture. Data collected over the fourth minute of each exercise trial were averaged and taken to reflect physiological steady-state wheelchair propulsion. From the VO2 (L/min), VCO2 (L/min) and respiratory exchange ratio (VCO2/VO2) the energy expenditure was determined using the formula proposed by Garby and Astrup [47].

### Data Analysis

**Figure 2:**
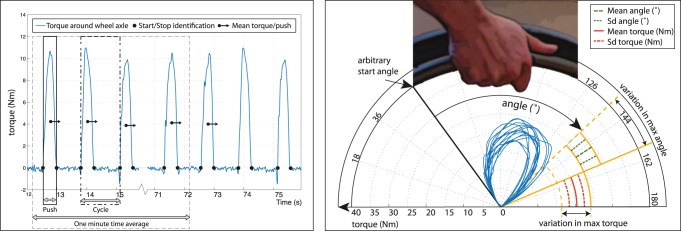
Two visualizations of propulsion kinetics. a) Time history of the torque signal showing the push identification, push-time, cycle-time, work per push, and mean torque. b) Alternative Polar plot of the torque against the angle for 12 pushes, showing the intra-individual variation in contact angle and maximum torque. Since no position data were recorded each push is started from the same arbitrary angle.

The data from the instrumented wheels were further analyzed using custom-written Matlab routines. To be certain of stable, steady-state propulsion, each last minute from the 4-min trials was used for the analysis. Per participant and trial, the torque (Nm) around the wheel-axle and the rotation angle (rad) were used to calculate the propulsion technique variables of interest. Individual pushes were defined as each period of continuous positive torque around the wheel axis with a positive minimum of at least 1 Nm. Over the identified pushes the propulsion technique variables were calculated and subsequently averaged over all pushes within the fourth minute of each practice trial per participant. The studied propulsion technique variables are defined in table 2 and figure 2. They were chosen based on their previously found association with mechanical efficiency (frequency, contact angle and negative work per cycle [42]) and two other variables were added because variability in them was expected to change (positive work per push and max torque/push (figure 2)).

**Table 2:** Definitions of propulsion technique variables.

Variable:	Description:	Equation:
Energy expenditure (W)	Calculated from the oxygen uptake and respiratory exchange ratioaccording to Garby and Astrup [47]	((4.94*RER+16.04)*(1000*VO2))/60
Gross mechanical efficiency (%)	The percentage of internal power used for external power deliveredat the wheels	Mean power output/Energyexpenditure
Frequency (push/min)	The number of complete pushes per minute.	(Cycletime)^−1^ ⋅ 60
Contact angle (°)	Angle at the end of a push minus the angle at the start.	Ø_end(i)_−Ø_start(i)_
Max Torque/push (Nm)	The maximum torque generated around the wheel-axle within a push	Max_start(i):end(i)_ (Tz)
Pos. Work/push (J)	The torque around the wheel-axle integrated over the contactangle of the push.	∑_ start(i):end(i)_ (Tz ⋅ ΔØ)
Neg. Work/cycle (J)	The torque around the wheel-axle integrated over the wheelrotation angle during the recovery phase	∑_end(i):start(i+1)_ (Tz ⋅ ΔØ)

Abbrevations: _start_(i), start of the current push (sample);_ end_(i), end of the current push (sample); Ø, angle (rad); Tz, torque around wheel axle (Nm).

### Statistics

Two groups (ISI and IFI), were formed based on a higher or lower than 10% relative increase in mechanical efficiency between the first and the third 4-min trial in the pretest, common for all interventions [42]. To replicate the results of the previous study for this smaller subset of participants the initial 12 minutes were pre-analyzed. Differences between the groups during the 12 min pretest were assessed on mechanical efficiency and propulsion technique using a repeated-measures Anova with the factors time, group and the interaction of time*group. Since not only the propulsion technique values, but also the variation therein is of interest, this process was repeated for the coefficient of variation, i.e. the percentage of standard deviation with respect to the mean. Significance level was set at p<0.05 for all statistical procedures.

Analysis of the inter-individual differences between the pre- and the posttest for the different learning trajectory groups was the main aim of this paper. To control for the different intervention types multi-level modeling was applied, [48]. The differences between the ISI and IFI were examined over all trials of the pre- and post-test to evaluate whether they were differently influenced by the longer practice period (i.e. an interaction effect of test*group). To correct for the different natures of the four interventions two extra terms were added to the model, namely ‘Duration’ (1-day or 3-wk) and ‘Variation’ (monotonous or variable). The model thus consisted of five terms: Test (pre = 0, post = 1), Learning group (ISI = 0, IFI = 1), Test*Learning group interaction, Duration (1 day = 0, 3 wk = 1) and Variation (no = 0, yes = 1). This model was applied to the dependent variables mechanical efficiency and selected propulsion technique variables (see table 2), as well as to the accompanying coefficient of variation of these outcome variables.

## Results

All participants were able to complete the protocol. The Optipush data (left side) were used for 35 participants and Smartwheel data (right side) were used for the other 4 participants. On average participants practiced at a power output of 17.6 W (s.d. = 4.2).

The differences between the ISI and IFI are presented for the first twelve minutes (repeated measures Anova) in table 3 and for the total 80 minutes (multi-level regression) in table 4. Changes in mechanical efficiency, propulsion technique and intra-individual variability for both groups over the first 12 and total 80 minutes are described below.

**Table 3:** Results of the repeated measures Anova on the three trials of the 12(n = 39).

Values pre-test Anova		mean 1	sd 1	mean 2	sd 2	mean 3	sd 3	p-Time	p-Group	p-Interaction
**Mechanical Efficiency (%)**	**ISI**	5.45	0.81	5.53	1.06	5.39	1.01	0.000	0.075	0.004
	**IFI**	4.38	0.94	4.99	1.03	5.25	1.20			
**Frequency (/min)**	**ISI**	68.22	13.30	69.27	12.59	68.42	14.96	0.000	0.910	0.002
	**IFI**	75.41	18.37	66.99	17.96	61.71	17.65			
**Frequency CV (%)**	**ISI**	8.81	4.87	7.51	4.53	5.94	3.02	0.075	0.010	0.918
	**IFI**	12.30	7.10	11.19	5.06	10.26	5.42			
**Neg work/cycle (J)**	**ISI**	−0.51	0.45	−0.50	0.62	−0.45	0.75	0.000	0.226	0.039
	**IFI**	−1.09	1.08	−0.84	1.06	−0.60	0.82			
**Neg work/cycle CV (%)**	**ISI**	116.07	175.98	78.17	60.21	82.90	50.02	0.800	0.892	0.036
	**IFI**	68.71	46.23	97.20	78.72	100.85	86.46			
**Contactangle (°)**	**ISI**	60.73	10.89	59.59	9.17	60.73	10.89	0.000	0.804	0.003
	**IFI**	53.29	10.89	61.31	12.61	63.60	13.18			
**Contactangle CV (%)**	**ISI**	12.72	4.73	9.85	1.80	9.02	1.89	0.000	0.008	0.784
	**IFI**	14.78	4.00	12.90	4.73	11.21	2.32			
**max Tz/push(Nm)**	**ISI**	13.84	2.59	13.55	2.37	13.58	2.62	0.526	0.825	0.933
	**IFI**	14.02	3.40	13.88	3.07	13.72	3.03			
**max Tz/push CV (%)**	**ISI**	18.37	3.10	16.25	2.95	15.37	3.23	0.000	0.004	0.600
	**IFI**	21.65	4.98	20.01	3.83	17.71	3.45			
**work/push (J)**	**ISI**	9.02	2.57	8.79	2.24	8.99	2.41	0.016	0.975	0.046
	**IFI**	8.13	2.72	9.22	2.99	9.37	2.88			
**work/push CV (%)**	**ISI**	21.96	4.47	19.09	2.75	17.58	3.98	0.000	0.004	0.435
	**IFI**	26.63	5.78	23.82	5.51	20.22	4.69			

P-values <.05 are interpreted as statistically significant results.

**Table 4:** Multi-level regression results for the 80-minute intervention for both the propulsion technique variables and their coefficient of variation (n = 39).

Values intervention MLWin		Cons	Time	p-Time	Group	p-Group	Interaction	p-Interaction	Duration	p-Duration	Variation	p-Variation
**Mechanical Efficiency (%)**	result	5.48	0.04	0.79	−0.55	0.04	0.90	0.00	0.58	0.06	−0.52	0.07
	s.e.	0.30	0.17		0.27		0.21		0.31		0.29	
**Frequency (/min)**	result	67.25	−15.19	0.00	0.82	0.85	−4.28	0.19	4.72	0.32	−0.94	0.83
	s.e.	4.74	2.60		4.15		3.26		4.79		4.45	
**Frequency CV (%)**	result	6.90	3.23	0.01	3.84	0.02	−3.50	0.01	−1.32	0.47	2.23	0.19
	s.e.	1.84	1.26		1.65		1.58		1.81		1.68	
**Neg work/cycle (J)**	result	−0.43	0.34	0.01	−0.39	0.02	0.41	0.01	−0.23	0.24	0.06	0.73
	s.e.	0.19	0.11		0.17		0.14		0.19		0.18	
**Neg work/cycle CV (%)**	result	91.54	142.40	0.00	−11.89	0.78	−105.48	0.04	−34.87	0.40	30.88	0.42
	s.e.	44.03	41.04		41.45		51.34		40.88		37.97	
**Contactangle (**°**)**	result	62.45	7.22	0.00	−1.43	0.67	7.96	0.01	−0.63	0.87	−4.07	0.25
	s.e.	3.78	2.12		3.32		2.69		3.78		3.50	
**Contactangle CV (%)**	result	10.14	−0.77	0.39	2.54	0.01	−1.88	0.08	−1.49	0.08	2.10	0.01
	s.e.	0.93	0.89		0.88		1.11		0.86		0.80	
**max Tz/push(Nm)**	result	11.87	0.94	0.02	0.76	0.39	−0.54	0.29	3.11	0.01	1.27	0.20
	s.e.	1.03	0.41		0.88		0.51		1.06		0.99	
**max Tz/push CV (%)**	result	16.36	−2.86	0.00	3.17	0.00	−1.21	0.26	−1.49	0.16	1.90	0.06
	s.e.	1.10	0.85		1.00		1.07		1.07		0.99	
**work/push (J)**	result	8.05	1.90	0.00	0.22	0.82	0.65	0.29	1.90	0.10	0.33	0.76
	s.e.	1.13	0.49		0.97		0.61		1.16		1.07	
**work/push CV (%)**	result	18.62	−3.26	0.01	4.26	0.00	−2.86	0.05	−1.67	0.23	3.39	0.01
	s.e.	1.44	1.17		1.32		1.46		1.38		1.28	

S.e. is the standard error of the multi-level model result. P-values <.05 are interpreted as statistically significant results.

### Gross Mechanical Efficiency

#### First 12 minutes of practice

Based on a 10% relative change in mechanical efficiency out of the 39 participants 13 were classified as ISI and 26 as IFI. Concomitant with this selection an interaction effect was found between the two groups based on the repeated measures Anova on the pretest, where the ISI already had a higher mechanical efficiency in the first 4-min trial than IFI (ISI 5.5% vs. IFI 4.4%, p<0.002).

#### Total 80 minutes of practice

**Figure 3:**
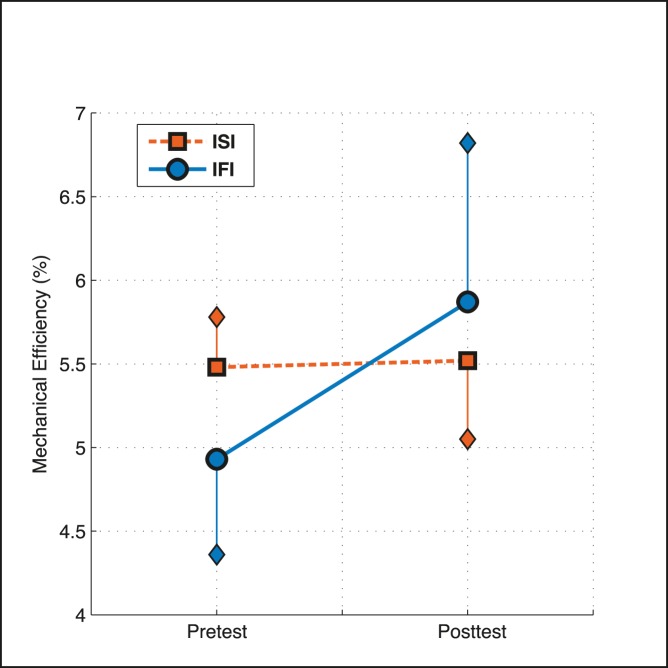
Effect of 80 minute practice time on the mechanical efficiency (mean and standard-error of multi-level model) for both groups between the pre- and posttest. While controlling for the nature of the intervention the n = 26 IFI (>10%) started with a lower mechanical efficiency but benefitted more from the intervention and had a higher mechanical efficiency in the posttest compared to the n = 13 ISI (≤10%).

Over the whole 80-minute intervention the interaction effect on mechanical efficiency remained consistent between the two groups over time (figure 3). Based on the multilevel regression analysis and controlling for the nature of the intervention the IFI, despite starting lower in the pretest, benefitted more from the intervention and had a significantly higher mechanical efficiency compared to the ISI at the posttest (ISI 5.5→5.5% vs. IFI 4.9→5.9%, p<0.001).

### Propulsion Technique

#### First 12 minutes of practice

Similar to the mechanical efficiency also an interaction effect was found during the pretest for the propulsion technique variables frequency, contact angle, work per push and negative work per cycle. For each of these variables the ISI had a significant better outcome in the first 4-min trial than the IFI but did not further improve in technique over the next 8 minutes. For the maximum torque per push no significant effect of trial, group or interaction within the two groups was found.

#### Total 80 minutes of practice

**Figure 4:**
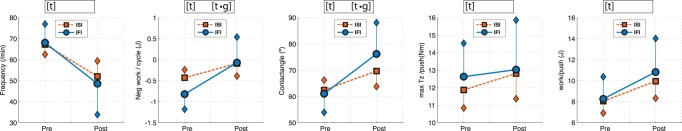
Effect of 80-minute practice on the propulsion technique (mean and standard-error of multi-level model) for the initially slow learners and the initially fast learners between the pre- and posttest. Contact angle and negative work per cycle showed an interaction effect for the groups over time. [t] = effect of time, [g] = effect of group, [t⋅g] = interaction effect of time with group.

For all propulsion technique parameters a significant effect of ‘Test’ was shown (figure 4). During the posttest participants of both groups had decreased their push frequency, reduced their amount of negative work, increased their contact angle, increased their maximum torque and finally increased their work per push.

Over the 80-minute intervention and after controlling for the nature of the intervention an interaction effect for ‘Test’*‘Learning Group’ was only found for the contact angle and the negative work per cycle (figure 4). The IFI increased significantly more in contact angle than the ISI and had a larger contact angle in the posttest (ISI 62.5°→69.7° vs. IFI 61°→76.2°, p<0.01). The IFI decreased significantly more in the negative work per cycle than the ISI (ISI 0.43 J→0.09 J vs. IFI 0.82 J →0.07 J, p<0.01).

### Intra-individual Variability

#### First 12 minutes of practice

During the pretest the IFI had a higher coefficient of variation for the frequency, contact angle, maximum torque and the work per push compared to the ISI (i.e. Anova effect of group). The coefficient of variation for the negative work per cycle showed an interaction effect; the ISI decreased, while the IFI increased in intra-individual variability.

#### Total 80 minutes of practice

**Figure 5:**
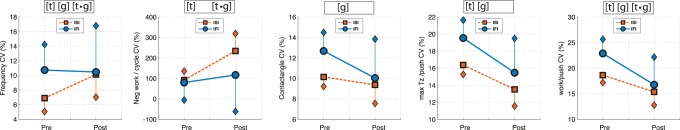
Effect of 80-minute practice on the intra-individual variability of the propulsion technique parameters (mean and standard-error of multi-level model) for the initially slow learners and the initially fast learners between the pre- and posttest. The frequency, contact angle, maximum torque per push and work per push showed an interaction effect for the groups over time. [t] = effect of time, [g] = effect of group, [t⋅g] = interaction effect of time with group.

A significant reduction in the coefficient of variations of frequency, maximum torque per push and work per push was shown for all participants in the posttest, i.e. a significant of ‘Test’ (figure 5). For the negative work the coefficient of variations significantly increased for all participants in the posttest.

Over the whole intervention a group effect was found for the coefficient of variations of the variables frequency, contact angle, maximum torque per push and work per push, where the IFI had higher coefficient of variations compared to ISI. An interaction effect was found for the coefficient of variations of frequency, negative work per cycle and positive work per push, but not all in the same direction. For frequency and negative work per cycle it were the ISI that increased more in the coefficient of variations, while for the work per push it were the IFI that decreased more.

## Discussion

Aim of the present study was to evaluate differences between individuals in learning low-intensity steady-state wheelchair propulsion on a motor-driven treadmill. Therefore two groups of learners were first identified, based on a higher (IFI) or lower (ISI) than 10% relative increase in mechanical efficiency, during the first twelve minutes of practice. Concomitant with this pretest difference in mechanical efficiency the ISI and IFI also differed in the change of propulsion technique and intra-individual variation during the first 12 minutes of practice. Over the total 80 minutes of low-intensity wheelchair-practice the two groups maintained different motor learning styles. Despite starting at a lower mechanical efficiency during the first minutes of practice, the IFI benefitted most of the given practice in terms of increased mechanical efficiency and better propulsion technique like an increased contact angle and reduced negative work [49].

Increased mechanical efficiency following practice is frequently found and thought to be indicative of motor learning [37], [38], [50]. Most of these studies have assessed motor learning by studying a single group as a whole. However, an indication for individual differences in the initial mechanical efficiency and change thereof was found in an earlier study, only analyzing the first 12 min of practice [42]. In the current study the effects of extended practice were studied, taking into account the individual differences in learning. The results indicate that the group of participants (IFI) that increased more in mechanical efficiency on a short term (during the pretest) also increased more over the long term, implying differences in the motor learning process between the two groups. Since all the interventions were low in intensity and total practice time, the changes in mechanical efficiency are presumably attributed to a changed propulsion technique instead of physiological adaptations expected from an extended high intensity dose [51].

The ISI started with better scores for the propulsion technique parameters, i.e. a larger contact angle, more work per push and less negative work than the IFI [49]. Yet, the IFI changed more in these parameters and in the twelfth minute they were on the same level as the ISI. For two variables, the contact angle and the negative work per cycle, this effect continued over the 80 minutes practice period. The contact angle of the IFI increased more and was higher in the posttest compared to the ISI. Since the work per push is the integration of positive torque around the axle over the angle through which it rotates, using a larger contact angle helps to increase the work per push and might help reduce peak forces and make the build up of force more gradual, possibly decreasing the risk of overuse injury [49], [52]–[54]. The IFI also reduced more in the negative work per cycle than the ISI. Because this negative work did not have to be compensated with positive work, in total less work is needed to maintain the same power output. As found in previous wheelchair learning studies, an effect of time was present for all propulsion technique variables, showing the effect of motor learning on propulsion technique for both learning groups [6]–[13].

Besides the means of the propulsion technique parameters, also the intra-individual variation in these parameters was studied. It was found that for all propulsion technique parameters the IFI had a significantly higher intra-individual variability during the 12-minute pretest than the ISI. Over the 80-minute practice the IFI continued to be more variable in frequency, contact angle, maximum torque per push and work per push. Possibly the IFI were more active in exploring different motor solutions, to find a more optimal pattern of coordination [28], [36], [37]. Besides the differences in intra-individual variability between the learning groups, a reduction in the intra-individual variation for both groups over time was found for the maximum torque and work per push. Contrary to our expectations, the reduction in intra-individual variation was not shown for the frequency, which would have been expected on basis of the decreased variability in work per push, since these two together should lead to an average constant power output over time in each trial, as required by the constant speed of the motor driven treadmill.

An earlier study on motor learning with the same practice dose and trial set up, but performed on a wheelchair ergometer, did not find reductions in the coefficient of variation of different propulsion technique variables [6]. Possibly, the higher freedom with the continued need to maintain a straight course and a mean fixed speed on the treadmill introduces extra elements to the learning task, which can be minimized over time [40].

**Figure 6:**
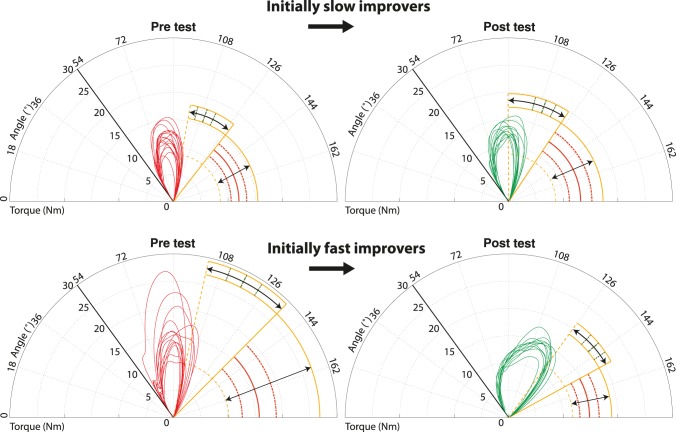
The first and last trial of a typical participant for both groups. The push-curves of the torque against the angle show the intra-individual variation in contact angle and maximum torque within a trial. The change over time because of practice is shown by the push-curves over time between the pre and posttest. Since no position data were recorded each push is started from the same arbitrary angle.

To illustrate the total change in propulsion technique, figure 6 shows the first and last trial of one typical participant for both groups. During the last 15 seconds for each push the torque around the wheel-axis is plotted against the wheel-angle in a polar plot. The difference in motor learning between the two participants can be visualized by both the amount of variation in the push-curves within a trial and the change of the push-curves over time. During the pretest the variation in peak torque and contact angle is much larger for the initially fast improver. Over the intervention the change of the shapes between the pre- and the posttest is much larger for the initially fast improver compared to the change of the initially slow improver. The post-test propulsion technique of the initially fast improver shows a larger contact angle and a much more gradual build up of torque than the initially slow improver, implying a more optimal propulsion technique [54].

Our findings suggest motor learning differences between able-bodied individuals regarding the acquisition of a low-intensity steady-state wheelchair propulsion skill. For rehabilitation practice it is important to appreciate that these motor learning differences between individuals exist, beside those differences caused by an individual’s specific impairment. Ideally, exercise programs with a focus on improving skill should be individually tailored to the motor learning style and capacity of the participants. Such a program may be beneficial to reduce external and internal mechanical loading of the upper limbs [55], [56], next to increasing the mechanical efficiency. Thus, the task load of handrim propulsion might be reduced and overuse injury may be prevented during early rehabilitation. More specific focus on motor learning is therefore necessary during the early rehabilitation of actual wheelchair–dependent persons, to further improve their rehabilitation outcomes.

In that sense the higher intra-individual variability found in the IFI gives some insight into the differences in motor learning strategy between the two groups. Further research on the link of inter-individual differences in intra-individual variation with motor learning processes might help to design more individualized and efficient rehabilitation programs. There is increasing evidence for an association between intra-individual variation and overuse injury [57]. A recent study showed that wheelchair-users with shoulder pain showed a lower intra-individual variability in peak resultant forces of the shoulder joint [58]. Possibly, the ISI in our study, showing a lower intra-individual variability over the 80 minutes of practice, are at a higher risk of developing overuse injury than the IFI. Thus, it may be beneficial from both a motor learning and an injury prevention perspective to develop interventions that try to elicit more intra-individual variation from the participants. In that sense the control variable Variation showed a significant increase in the coefficient of variation in contact angle and work/push, giving a possible direction for future research on increasing intra-individual variation.

Several limitations should be taken into account when interpreting the results of the current study. First, the different interventions were not originally intended to discriminate between the two learning groups, but were focused on other motor learning related research questions. Using a multi-level model we have tried to correct for practice variability and total duration to make a comparison between the different possible interventions. Fortunately the ratio between the initially slow and fast learners was pretty comparable for the different interventions (table 1). Secondly, all subjects practiced in a standardized wheelchair without adjustments for the participant’s anthropometry. It could be that this setup gave more room for improvement for some participants compared to others. Finally, the groups were split on a pre-set criterion of 10% increase in mechanical efficiency during the pre-test. This is a first attempt to identify different groups of learners in a cyclic steady-state low-intensity wheelchair propulsion intervention. However, whether there are only 2 groups of learners or more cannot be certain from the current research. Perhaps in the future more data-driven methods like cluster analysis can be used to explore what kind of groups can be logically put together [22].

## Conclusion

The IFI, about two thirds of the able-bodied novice participants, started the pretest with a lower mechanical efficiency and a less optimal propulsion technique. However already during the pretest the IFI learned more and this effect continued over the total 80 minutes of practice, while controlling for differences in the practice format. Eventually the IFI benefitted more from the given practice compared to the ISI and learned a better propulsion technique, performed at a higher mechanical efficiency. Over the given practice the IFI had a higher intra-individual variability in the pre and posttest. Possibly this higher variability relates to the increased motor learning of the IFI. Individual motor learning differences are important to take into account for rehabilitation programs. Further exploration of the common characteristics of different types of learners will help to better tailor rehabilitation to the specific needs of wheelchair dependent persons.
